# Biologically Synthesized Gold Nanoparticles Ameliorate Cold and Heat Stress-Induced Oxidative Stress in *Escherichia coli*

**DOI:** 10.3390/molecules21060731

**Published:** 2016-06-04

**Authors:** Xi-Feng Zhang, Wei Shen, Sangiliyandi Gurunathan

**Affiliations:** 1 College of Biological and Pharmaceutical Engineering, Wuhan Polytechnic University, Wuhan 430023, China; zhangxf9465@163.com; 2 Key Laboratory of Animal Reproduction and Germplasm Enhancement in Universities of Shandong, College of Animal Science and Technology, Qingdao Agricultural University, Qingdao 266109, China; shenwei427@163.com; 3 Department of Stem Cell and Regenerative Biology, Konkuk University, Seoul 143-701, Korea

**Keywords:** *Escherichia coli*, gold nanoparticles, heat stress, cold stress, oxidative stress, antioxidants

## Abstract

Due to their unique physical, chemical, and optical properties, gold nanoparticles (AuNPs) have recently attracted much interest in the field of nanomedicine, especially in the areas of cancer diagnosis and photothermal therapy. Because of the enormous potential of these nanoparticles, various physical, chemical, and biological methods have been adopted for their synthesis. Synthetic antioxidants are dangerous to human health. Thus, the search for effective, nontoxic natural compounds with effective antioxidative properties is essential. Although AuNPs have been studied for use in various biological applications, exploration of AuNPs as antioxidants capable of inhibiting oxidative stress induced by heat and cold stress is still warranted. Therefore, one goal of our study was to produce biocompatible AuNPs using biological methods that are simple, nontoxic, biocompatible, and environmentally friendly. Next, we aimed to assess the antioxidative effect of AuNPs against oxidative stress induced by cold and heat in *Escherichia coli*, which is a suitable model for stress responses involving AuNPs. The response of aerobically grown *E. coli* cells to cold and heat stress was found to be similar to the oxidative stress response. Upon exposure to cold and heat stress, the viability and metabolic activity of *E. coli* was significantly reduced compared to the control. In addition, levels of reactive oxygen species (ROS) and malondialdehyde (MDA) and leakage of proteins and sugars were significantly elevated, and the levels of lactate dehydrogenase activity (LDH) and adenosine triphosphate (ATP) significantly lowered compared to in the control. Concomitantly, AuNPs ameliorated cold and heat-induced oxidative stress responses by increasing the expression of antioxidants, including glutathione (GSH), glutathione S-transferase (GST), super oxide dismutase (SOD), and catalase (CAT). These consistent physiology and biochemical data suggest that AuNPs can ameliorate cold and heat stress-induced oxidative damage in *E. coli.* Our results indicate that AuNPs may be effective antioxidants. However, further studies are needed to confirm the role of AuNPs as antioxidative agents, as well as their mechanism of action.

## 1. Introduction

Gold nanoparticles (AuNPs) are being used in biomedical applications such as cancer therapy (as drug carriers), cellular imaging, molecular diagnosis, and targeted therapy, and as contrast agents, photothermal agents, and radiosensitizers. Their usefulness is due to their stability and unique optical, magnetic, electronic, oxidation resistance, and structural properties, as well as to their structure, composite, and shape [1,2]. AuNPs, among other metallic nanoparticles, have attracted much interest in cancer research, due to their facile synthesis and surface modification, strongly enhanced and tunable optical properties, and excellent biocompatibility. Another major advantage of using AuNPs is that their preparation is simple, with high quality and high yield, and their size can be controlled [2,3].

Several methods have been established for synthesis of AuNPs, including physical, chemical, and biological methods. Chemical methods are the most widely and traditionally used, and rely on various reducing agents such as hydrazine and sodium borohydride [1,3]. Although the physical and chemical methods appear to be simple, they have numerous disadvantages, such as the necessity for high temperatures and pressures and toxic chemicals; most importantly, they can cause the particles to become unstable or aggregate upon interaction with biological media or biomolecules [3,4,5]. Furthermore, they require a long centrifugation process to produce multi-shaped or controlled-size particles. Recently, biological methods have shown much promise in producing nanomaterials using an environmentally friendly approach and biological material as a reducer, along with stabilizing agents to produce shape- and size-controlled particles for mass production with high yield, solubility, reproducibility, and biocompatibility [1,3]. Several microorganisms and plant extracts have been used for synthesis of AuNPs, including *Lactobacillus* strains [6], *Bacillus licheniformis* [7], *Brevibacterium casei* [8], *Aspergillus fumigatus* [9], edible mushroom extract [10], and medicinal mushroom extract [3]. Although several reports have demonstrated synthesis of AuNPs using a variety of biological materials, exploration of novel biological materials is warranted, because every living entity has unique surface functionalization properties that can provide significant biocompatibility to targeting agents.

Stress response in bacteria is an essential and indispensable protection against various external stimuli. It leads to changes in the physiological state of the bacterial culture itself [11]. Biological structures in bacteria, yeasts, plants, animals, and humans are frequently disturbed by various stresses, such as ultraviolet radiation, pH, salinity, heat, and cold [12]. Cold stress responses in bacteria occur in two phases: a transient shock response and a continuous acclimation response [12]. When bacteria are exposed to low temperatures, they develop membrane modifications to maintain the membrane fluidity and structural integrity of macromolecule assemblies such as proteins and ribosomes [13,14,15,16]. Changes in temperature are a kind of stress that triggers protective mechanisms by gene expression and synthesis of a specific set of proteins known as heat shock proteins (HSPs) [17,18]. Generally, high temperatures create more interruption of the cellular metabolism, and require a higher level of stability of enzymes and other macromolecules to maintain cell survival [19].

Recently, several studies have reported that environmental agents such as UV radiation, nanomaterials, toxic agents, and heat and cold stress stimulate production of ROS in bacterial cells. ROS are reactive byproducts formed by the partial reduction of molecular oxygen, which arises when the concentration of active oxygen increases to a level that exceeds the cell’s defense capacity [20]. The biological targets for these highly reactive oxygen species are DNA, RNA, proteins, and lipids. Among the biological molecules, lipids are major targets during oxidative stress [21]. In nature, living entities must face unfavorable conditions during their lives and reproductive processes [22]. For instance, bacteria have evolved various adaptive conditions by the synthesis of new proteins and they increase the activity of antioxidant systems under various stress conditions including temperature, pH, oxygen concentration, and solar radiation. When high levels of ROS are present, several defense systems, such as superoxide dismutase, catalase, and peroxidase, maintain a steady-state level of ROS in order to protect the cell [23].

AuNPs have been used to treat diseases such as smallpox, skin ulcers, syphilis, and measles, and show anti-leishmanial and antimalarial activity [24,25]. Interestingly, gold compounds are used as anti-inflammatory agents due to their ability to inhibit expression of NF-kappaB and subsequent inflammatory reactions [26]. Recently, AuNPs were found to exhibit antioxidative properties by inhibiting the formation of ROS, scavenging free radicals, and increasing antioxidant defense enzymes in diabetes-induced mice [27]. Although biomedical research has focused extensively on AuNPs, to our knowledge, no studies have reported the protective role of AuNPs in cold or heat-induced oxidative stress in bacteria.

Generally, *Escherichia coli* is considered a suitable model for stress responses, and is a particularly valuable model for exploring how single-celled organisms respond to environmental stresses [28]. In addition, it offers several advantages over other models due to the relative ease of modifying and manipulating it to study the mechanisms of oxidative stress. Furthermore, the absence of intracellular organelles makes possible a more accurate quantization of oxidants generated in the reactions [29]. Therefore, we have chosen *E. coli* as a model organism to elucidate the antioxidative properties of AuNPs in bacteria. To date, the protective effect of gold nanoparticles against cold- or heat-induced stress in bacteria is obscure. Therefore, the first aim of this study was the synthesis and characterization of gold nanoparticles using a novel probiotic bacterium called *Bacillus clausii*. The second aim was to investigate the effect of AuNPs on cold and heat-induced oxidative stress in *E. coli*.

## 2. Results and Discussion

### 2.1. Synthesis and Characterization of AuNPs

**Figure 1:**
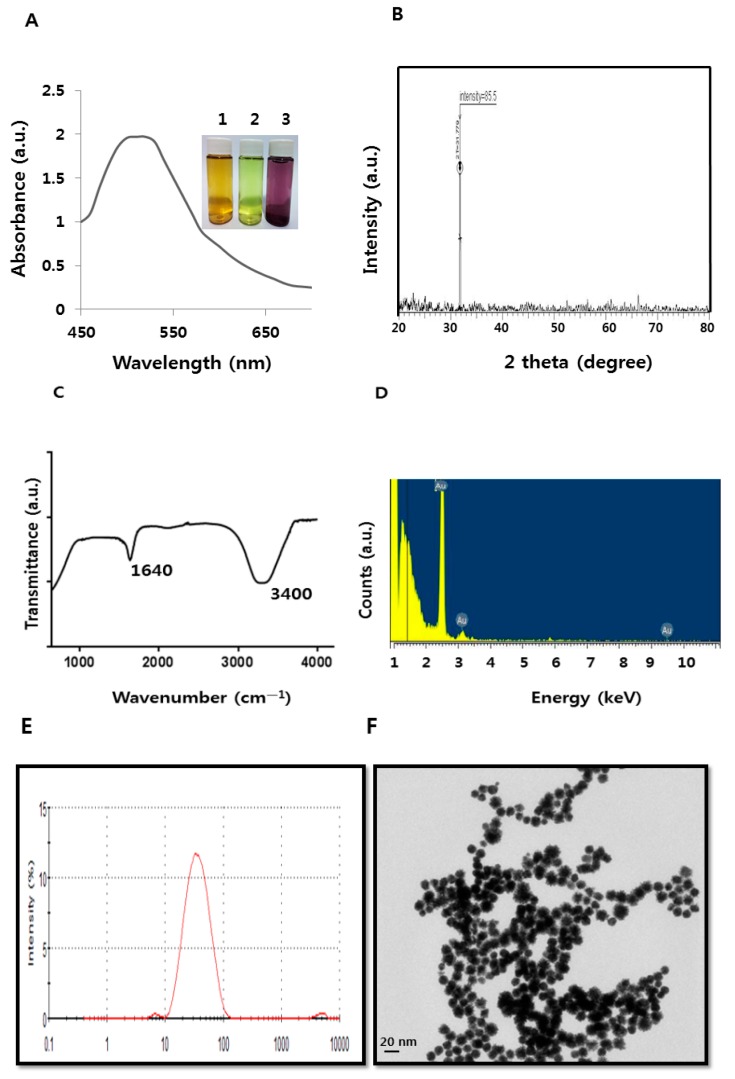
Synthesis and characterization of gold nanoparticles (AuNPs) using *Bacillus clausii*. (**A**) The absorption spectrum of AuNPs synthesized by *B. clausii* culture supernatant. The inset shows tubes containing samples of the *B. clausii* supernatant after exposure to 1 mM aqueous HAuCl_4_ for 2 h (1), supernatant (2), HAuCl_4_ (3) HAuCl_4_ plus supernatant. The color of the solution turned from pale yellow to purple after 2 h of reaction time, indicating the formation of AuNPs; (**B**) X-ray diffraction spectrum of AuNPs; (**C**) FTIR spectra of AuNPs; (**D**) EDX spectra of AuNPs; (**E**) Size distribution analysis of AuNPs by DLS; (**F**) Size and surface morphology analysis of AuNPs by TEM.

Whether they are prokaryotes or eukaryotes, biological organisms are known to defend themselves under unfavorable or extreme stress conditions such as extreme cold or heat. Fortunately, microbiologists and nanobiotechnologists can exploit microbes for the fabrication of nanomaterials or metal nanoparticles, as opposed to the environmentally toxic chemical method [3,4,5,30]. In this study, we explored a novel bacterium, *B. clausii*, for synthesis of AuNPs by the addition of selected culture supernatant to 1 mM aqueous HAuCl4 at 40 °C. Within 2 h of incubation, the reaction mixture turned from yellow to a dark purple color (Figure 1A inset). This color formation is dependent on the excitation of surface plasmon vibrations of AuNPs [3,31,32]. It is primary evidence for the synthesis of AuNPs.

After primary characterization by observing visible color change, we performed a UV-vis measurement. The ultraviolet-visible (UV-vis) spectrum shows maximum absorption at 520 nm, which is the typical surface plasmon resonance (SPR) band of AuNPs (Figure 1A). Observation of this peak, assigned to a surface plasmon, is well documented for various metal nanoparticles with sizes ranging from 2 to 100 nm [33]. The diffraction peaks of prepared AuNPs shown at 31.8 °C corresponded to the (111) planes, respectively (Figure 1B). No extra peak was observed in the diffraction peaks, indicating that the biologically prepared AuNPs were highly purified without any contamination [3]. Next, FTIR analysis was performed to further show that biomolecules were responsible for synthesis and stabilizing of AuNPs by the culture supernatant of *B. clausii*. The AuNPs synthesized by the culture supernatant yielded strong bands at 1640 cm^−1^ (Figure 1C). These bands correspond to the amide I, II, and III bands of polypeptides/proteins, and are consistent with previous reports [3,34]. These peaks also represent the vibrational modes of C=C double bonds of these molecules. Generally, the bands represented in the region between 1550 and 1650 cm^−1^ correspond to a secondary amine NH bend [35]. In addition, a prominent band was observed at 3400 cm^−1^, corresponding to the carbonyl and hydroxyl functional groups in alcohols and phenol derivatives [3,5]. Overall, the FTIR results show that surface capping of AuNPs synthesized by the water-soluble culture supernatant of bacteria is predominantly by proteins, which are favored in the biosynthesis of AuNPs.

Next, we examined EDS to identify the elemental composition of biologically prepared AuNPs. As shown in Figure 1D, the EDS profile shows a strong gold signal along with weak oxygen and carbon peaks, which may have originated from biomolecules of culture supernatant bound to AuNPs surfaces. The strong signal clearly indicates that the particle composition is gold, and indicates that the biomolecules of bacteria bound to the surface of the AuNPs originated from biological systems. The results of our experiments align with previous reports demonstrating synthesis of AuNPs using various biological materials such as chloroblasts [36], plant extracts [37], *Bacillus flexus* [32], and fungal extracts [3] as reducing and stabilizing agents.

Determination of monodispersity, uniformity, size, and surface morphology of prepared AuNPs is essential and indispensable for evaluation of the biological properties of synthesized AuNPs in bacterial or any cell line studies. Therefore, we performed size distribution and surface morphology analysis by DLS and TEM, respectively. DLS can provide a particular size distribution range as compared to TEM. As shown in Figure 1E, DLS analysis showed that the prepared AuNPs had an average size of 30 nm, which was larger than the size measured by TEM due to Brownian motion. The TEM results clearly suggest that the particle size is 20 nm, with a narrow size distribution, and that all the particles are significantly spherical in shape (Figure 1F), which precisely matches the profile of *B. flexus* [32]. However, other *Bacillus* species, such as *B. licheniformis*, also produce 10–100 nm nanocubes [31]. Previously, we showed that alpha amylase from *B. licheniformis* produced particles with an average size of 10–50 nm [8]. Fibrinolytic enzymes from *B. cereus* produced an average particle size of 20 nm [38]. *Shewanella oneidensis* produced discrete extracellular spherical gold nanocrystallites with an average size of around 12 nm [5].

### 2.2. Effect of AuNPs on E. coli

**Figure 2:**
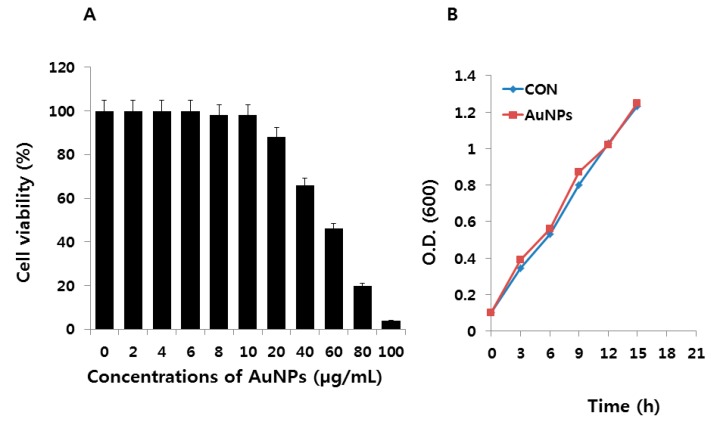
Effect of different concentrations of AuNPs and time on *E. coli* cell viability and growth. (**A**) *E. coli* cells were treated with AuNPs at various concentrations (from 0 to 100 μg/mL) for 24 h, and cell viability was determined by the CFU method. Data are shown as means and standard deviations calculated from three independent experiments; (**B**) Cells were incubated with 1 μg/mL of AuNPs. Samples were withdrawn at different points of growth, and the cells were centrifuged, washed with distilled water, and analyzed for growth at 600 nm. Data are shown as means and standard deviations calculated from three independent experiments.

In order to determine the antioxidative properties of AuNPs under cold- and heat-induced oxidative stress in *E. coli*, we first performed a cell viability assay to determine the biocompatibility effect of AuNPs on *E. coli*. AuNPs were added to LB medium at different concentrations. *E. coli* was exposed to 0–100 μg/mL for 12 h at 37 °C. At selected times, 100 μL of medium was sampled, diluted, and cultured on LB agar plates, and its viability evaluated. CFU methods are accurate and conventional techniques for determining bacterial numbers with little influence from NPs [39]. CFU data were converted to percentages of cell viability, as shown in Figure 2A. The cell viability was decreased as the concentrations of the AuNPs increased from 20 to 100 μg/mL. At 100 μg/mL of AuNPs, an approximately 96% reduction of cell viability as compared to the control sample was caused, as these concentrations represent the MIC values. The presence of AuNPs had no significant effect on cell viability up to the tested concentrations of 10 μg/mL, and exhibited biocompatibility with *E. coli*. Although the biocompatible effect was observed up to 10 μg/mL, we chose lower concentrations, such as 1 μg/mL, for further experiments. It is important to choose lower concentrations to avoid any false-negative or false-positive misinterpretation of the results. Next, we evaluated whether a low concentration of AuNPs could influence the growth of cells under long periods of incubation. The cells were treated with AuNPs (1 μg/mL) and incubated for 21 h. The results of this time-dependent experiment also show that AuNPs had no significant effect on cell viability (Figure 2B). Therefore, we selected 1 μg/mL for further experiments.

### 2.3. AuNPs Ameliorate Cold- and Heat-Induced Cell Death

**Figure 3:**
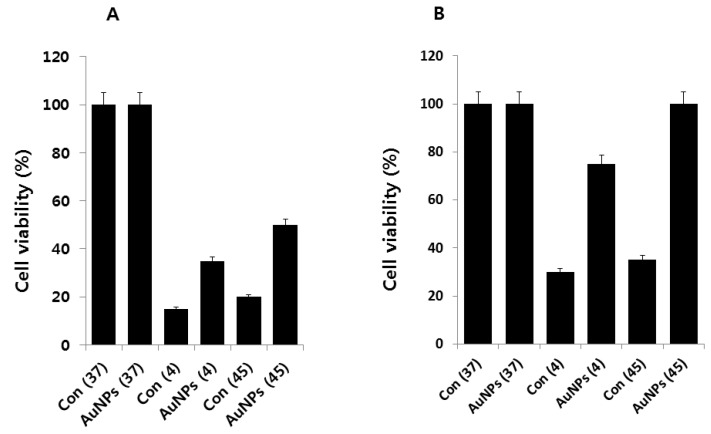
Effect of AuNPs on cold and heat stress-induced loss of viability. (**A**) *E. coli* cells were pre-incubated with AuNPs (1 μg/mL) for 1 h, stressed at 4 °C or 45 °C, and subsequently recovered for 6 h at 37 °C. Cell viability rates were determined by the colony-counting method and expressed as a percentage of the control; (**B**) *E. coli* cells were pre-incubated with AuNPs (1 μg/mL) for 1 h, stressed at 4 °C or 45 °C, and subsequently recovered for 12 h at 37 °C. Cell viability rates were determined by the colony-counting method and expressed as a percentage of the control. Data are shown as means and standard deviations calculated from three independent experiments. AuNP-treated groups showed statistically significant differences from the control group by Student’s *t*-test (*p* < 0.05).

Cold stress was induced by lowering the culture temperature from 37 to 4 °C, and heat stress was induced by raising the culture temperature from 37 to 45 °C for 2 h. The cells were pre-incubated with AuNPs for 1 h before introduction to either cold or heat stress. Cells without AuNPs were used as controls. An additional control was kept at 37 °C with and without AuNPs, because 37 °C is the optimal temperature for *E. coli* growth. After the cells were exposed to the target temperatures in the presence or absence of AuNPs, they were brought to 37 °C. A viability assay was performed after 6 and 12 h. The results clearly suggest that both cold and heat stress-induced cell death; however, the cells pretreated with AuNPs were able to rescue the cell death caused by cold and heat stress by at least 15% and 30% at 6 h, and 45% and 65% at 12 h duration, respectively (Figure 3A,B). As expected, by increasing the time of incubation at 37 °C after cold or heat stress, more cell viability was recovered in the presence of AuNPs. Interestingly, the rescue effect was more effective for heat stress than for cold. We found that 1 μg/mL of AuNPs suppressed the loss of viability, which suggests that AuNPs are highly protective to cells. The survival rate of heat stress-induced *E. coli* was 65% in the presence of AuNPs, even at a concentration of 1 μg/mL. In comparison, cold stress caused a 45% survival rate at up to 1 μg/mL of AuNPs, and AuNPs showed less protection against cold- than against heat-induced stress. These data showed that the protective effect of AuNPs is more pronounced against heat stress than against cold.

### 2.4. Effect of AuNPs on Cold and Heat Stress-Induced ROS

**Figure 4:**
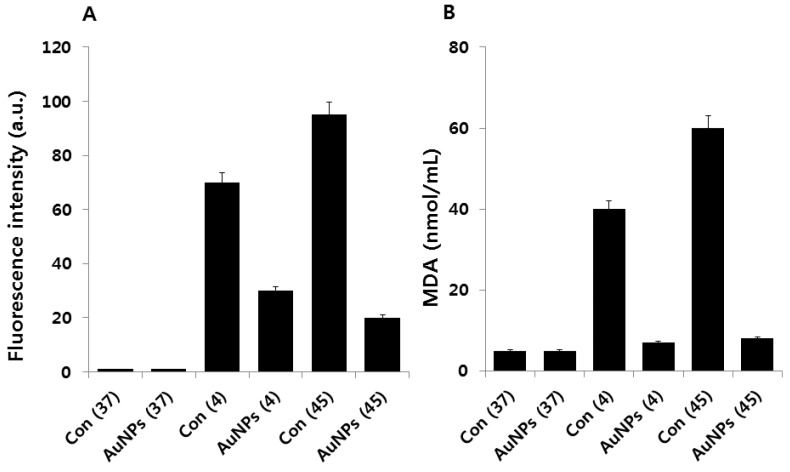
Effect of AuNPs on cold and heat stress-induced ROS generation. (**A**) *E. coli* cells were pre-incubated with AuNPs (1 μg/mL) for 1 h, stressed at 4 °C or 45 °C, and subsequently recovered for 12 h at 37 °C. ROS generation was measured by DCFDA. Data are shown as means and standard deviations calculated from three independent experiments. AuNP-treated groups showed statistically significant differences from the control group by Student’s *t*-test (*p* < 0.05); (**B**) *E. coli* cells were pre-incubated with AuNPs (1 μg/mL) for 1 h, stressed at 4 °C or 45 °C, and subsequently recovered for 12 h at 37 °C. The MDA level was measured by TABARS assay. Data are shown as means and standard deviations calculated from three independent experiments. AuNP-treated groups showed statistically significant differences from the control group by Student’s *t*-test (*p* < 0.05).

Heat stress is a major environmental stress that limits bacterial growth, metabolism, and reproduction. The physiology of bacterial response is dependent on temperature, and low temperatures may either allow growth to continue or, if sufficiently low, eventually lead to cell death [12]. ROS are often generated, depending on a variety of cellular conditions such as pH and temperature, and cell exposure to stress can lead to a drastic increase in ROS production [40]. In bacteria, proteins are major biological targets for oxidative damage within cells due to their high abundance and rapid rates of reaction with ROS [29,41,42]. Oxidative stress is known to damage the cell membrane, proteins, DNA, and intracellular systems such as the respiratory system [43]. In the present study, we measured the level of ROS using DCFDA in cold and heat stress-treated *E. coli* as well as *E. coli* that were pre-treated with AuNPs. As shown in Figure 4A, after 2 h incubation, a significant increase in ROS was detected in cold and heat stress-induced *E. coli*, but not in the control group. Interestingly, AuNP-pretreated cells showed clear downregulation of ROS production.

It has shown that high levels of free radicals or ROS can inflict direct damage on lipids. Lipid peroxidation is nothing but free radicals or non-radical species attacking lipids containing carbon–carbon double bonds [44]. MDA has been widely used as a convenient biomarker for lipid peroxidation of omega-3 and omega-6 fatty acids because of its facile reaction with thiobarbituric acid (TBA) [45]. Several studies have reported that MDA production is directly related to the concentration of generated ROS in the system [46]. To substantiate the relationship between ROS and MDA content in cold and heat stress-induced *E. coli*, we measured MDA content. As shown in Figure 4B, the levels of MDA were significantly higher in cold and heat stress-induced *E. coli* than in the controls. It is also noteworthy that the amount of MDA was 6- to 7-fold reduced by pretreatment with AuNPs relative to the stress-induced *E. coli.* These results suggest that the inhibition of bacterial growth due to cold or heat stress is attributable to ROS formation. Taken together, these results indicate that cell death is mediated by stress-activated ROS production, which creates an imbalance between oxidants and antioxidants in the cellular system.

### 2.5. Effect of AuNPs on Metabolic Activity

**Figure 5:**
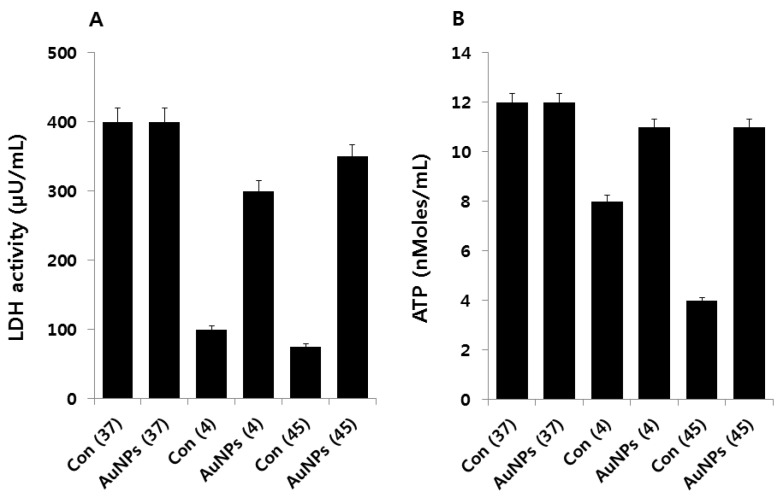
Effect of AuNPs on LDH activity and ATP levels. (**A**) *E. coli* cells were pre-incubated with AuNPs (1 μg/mL) for 1 h, stressed at 4 °C or 45 °C, and subsequently recovered for 12 h at 37 °C. LDH activity was determined by measuring the reduction of NAD^+^ to NADH and H^+^ during the oxidation of lactate to pyruvate. Data are shown as means and standard deviations calculated from three independent experiments. AuNP-treated groups showed statistically significant differences from the control group by Student’s *t*-test (*p* < 0.05); (**B**) *E. coli* cells were pre-incubated with AuNPs (1 μg/mL) for 1 h, stressed at 4 °C or 45 °C, and subsequently recovered for 12 h at 37 °C. The ATP level was determined by measuring luminescence levels and comparing them to an ATP standard curve. Data are shown as means and standard deviations calculated from three independent experiments. AuNP-treated groups showed statistically significant differences from the control group by Student’s *t*-test (*p* < 0.05).

To corroborate the oxidative stress-induced damage to the respiratory system of the cells, LDH activity was measured. As compared to the *E. coli* in the control group, the stress-activated cells exhibited decreased levels of LDH, whereas LDH activity increased in cells treated with AuNPs (Figure 5A). LDH activity in stress-treated cells was significantly lower than that of the control group. These results indicate that ROS formed by cold and heat stress inhibit LDH, which in turn inhibits cell growth, viability, and reproduction. These results are consistent with earlier reports demonstrating LDH denaturation under stress conditions [47], where LDH was studied as a representative denatured protein under oxidative and heat stress conditions [48]. The inactivation and inhibition of LDH leads to increased leakage of proteins and other macromolecules.

Subsequently, we measured the level of ATP in cold and heat stress-induced *E. coli* and AuNPs-pretreated samples. ATP is a vital molecule for many biological functions, including survival, growth, and replication, and is involved as a signaling molecule [49]. We measured ATP levels in the cultures of all tested samples. The ATP levels in cold and heat stress-induced samples were lower than in the controls. Surprisingly, the level of ATP was not highly influenced by cold stress; conversely, heat stress influenced ATP levels dramatically. The ATP level in the supernatant of the AuNPs-pretreated samples was noticeably similar to the level in the controls (Figure 5B). The amelioration effect of AuNPs on extracellular ATP release needs further characterization; nevertheless, the rescue effect of AuNPs on ATP might affect additional functions in bacterial physiology in addition to its role as an energy supplier [49]. Overall, cold and heat stress play an important role in the metabolic activity of *E. coli*, which ultimately controls the growth and reproduction of these bacterial cells.

### 2.6. Effect of AuNPs on Leakage of Proteins and Sugars

**Figure 6:**
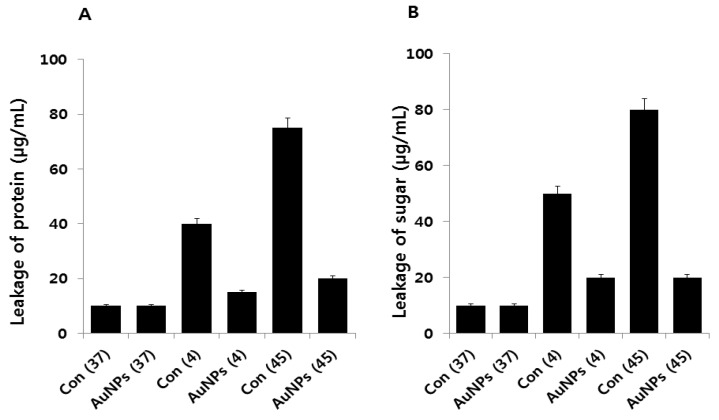
Effect of AuNPs on leakage of proteins and sugars. (**A**) *E. coli* cells were pre-incubated with AuNPs (1 μg/mL) for 1 h, stressed at 4 °C or 45 °C, and subsequently recovered for 12 h at 37 °C. Leakage proteins were measured using the Bradford assay; (**B**) The concentrations of sugars were measured. Data are shown as means and standard deviations calculated from three independent experiments. Treated groups showed statistically significant differences from the control group by Student’s *t*-test (*p* < 0.05).

Protein and sugar leakage from cells is a characteristic feature of heat damage in microorganisms. We observed that both heat and cold stress could enhance protein leakage by increasing the membrane permeability of *E. coli* cells (Figure 6A). Leakage from cells treated with cold temperatures was significantly higher than that of cells in the control group. Interestingly, cells pretreated with AuNPs showed significantly lower leakage than stress-induced cells did. Notably, our observations indicate that higher amounts of proteins leaked under stress conditions, suggesting that these increased the sensitivity of the cells more than the control or AuNP pretreatment.

Next, the amount of sugars released into the cell suspension after each stress treatment was analyzed (Figure 6B). Cold and heat stress significantly affected the amount of sugar leakage as compared to the control. However, the amount of sugar leakage from cells treated with heat stress substantially differed from the control. These results indicate that in most of the stress-induced cells, intracellular materials were released into the cell suspension. The sugar release pattern under the two stress conditions was the reverse of the sugar release pattern by AuNPs; the amount of leaked sugars under both stress conditions was found to be much lower when AuNPs were present. As reported earlier, high temperatures or freezing may produce changes in the outer cell layers of bacteria, leading to morphological and structural changes (blebbing) and loss of outer membrane lipopolysaccharides (LPS) [50,51]. However, the protective mechanism of the action of AuNPs in the leakage of proteins and sugars is still unknown.

### 2.7. Impact of AuNPs on GSH Levels

**Figure 7:**
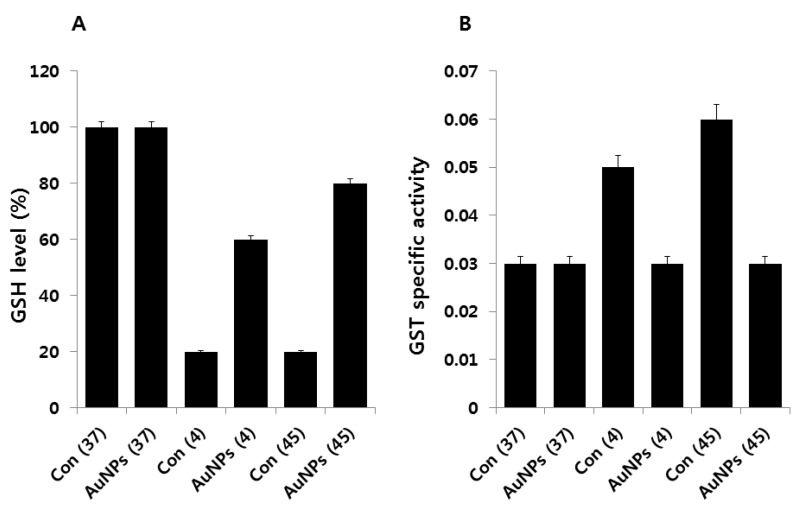
Effect of AuNPs on GSH and GST activity. (**A**) *E. coli* cells were pre-incubated with AuNPs (1 μg/mL) for 1 h, stressed at 4 °C or 45 °C, and subsequently recovered for 12 h at 37 °C. The amount of GSH was measured enzymatically in the clear supernatant based on the reduction of 5,5′-dithiobis-(2-nitrobenzoic acid) by the GSH reductase system; (**B**) GST activity was determined as described in the Materials and Methods section. Data are shown as means and standard deviations calculated from three independent experiments. Treated groups showed statistically significant differences from the control group by Student’s *t*-test (*p* < 0.05).

We hypothesized that if cold or heat exerts oxidative stress, as evidenced by our results, this should affect cellular antioxidant metabolites such as glutathione (GSH). The tripeptide, GSH, and other thiols are major cellular antioxidants [52,53]. We measured GSH levels under two different stress conditions. Cells exposed to stress showed decreased levels of GSH; however, AuNPs pretreatment caused an immediate increase in GSH levels (Figure 7A). Furthermore, this was consistent with the results of this and other studies, indicating that heightened levels of oxidative stress are responsible for reduced levels of GSH in stress-exposed *E. coli*. Cellular levels of GSH began to recover coincident with reduction in internal oxidative stress and cellular recovery. These events also coincided with the induction of proteins likely to be involved in countering oxidative stress. Our findings are consistent with previous findings that in cells preadapted to low temperatures and grown at 20 °C, the concentration of GSH was 2.4 times lower than in cells grown at 37 °C [54]. However, the detailed mechanism of the effect of AuNPs on GSH levels must be understood in order to establish any protective role for glutathione in defending against cold and heat stress.

To corroborate the results of our GSH analysis, we measured the activity of GST. GST is an enzyme whose activity is closely related to GSH; it is primarily involved in detoxification processes. Our aim was to measure GST activity to correlate the level of GSH concentration with GST. Total specific GST activity was measured, and the results revealed a distinct response for cold and heat stress (Figure 7B). Total GST activity increased from 0.03 units to 0.05 and 0.06 in cold and heat stress, respectively. When *E. coli* was grown in the presence of AuNPs, its GST activity was similar to that of the control. These results suggest that AuNPs maintain levels of both GSH and GST activity. Peters *et al*. reported that both glutathione reductase (GR) and glutathione S-transferase (GST) activity, together with the antioxidant compound, GSH, play major roles in the tolerance of a *Pseudomonas aeruginosa* strain to different herbicides [55]. The same scenario could exist in cold- and heat-induced oxidative stress in *E. coli*.

### 2.8. Effect of AuNPs on SOD and Catalase Activity

**Figure 8:**
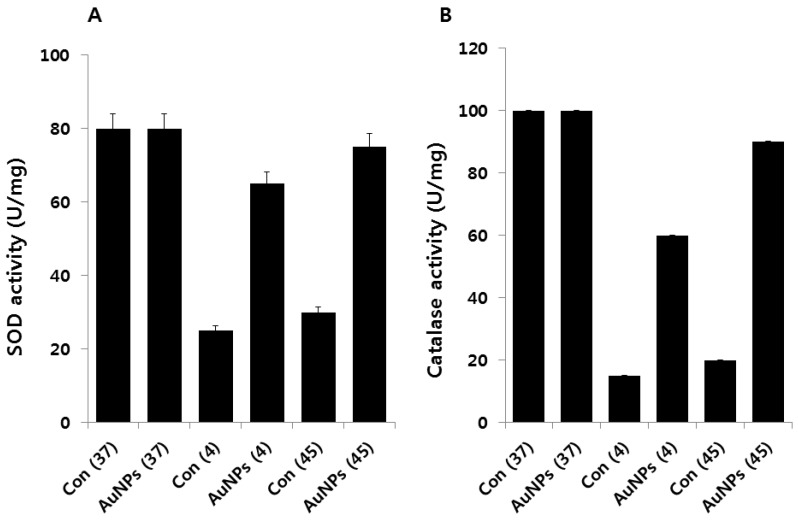
Effect of AuNPs on SOD and CAT activity. *E. coli* cells were pre-incubated with AuNPs (1 μg/mL) for 1 h, stressed at 4 °C or 45 °C, and subsequently recovered for 12 h at 37 °C. SOD (**A**) and CAT activity (**B**) were measured as described in the Materials and Methods section. Data are shown as means and standard deviations calculated from three independent experiments. The treatment groups showed statistically significant differences from the control group by Student’s *t*-test (*p* < 0.05).

Exposure of living cells to moderate or high temperatures produces a small, selective number of highly conserved proteins called heat shock proteins [17]. SOD is known to be induced by oxidative stress imposed by dioxygen. Previous studies show that heat shock induces increased biosynthesis of MnSOD by *E. coli* [56]. To estimate the contribution of antioxidant systems to bacterial response to cold and heat stress, we examined the activity of SOD and CAT in the presence and absence of AuNPs. The obtained data suggest that both cold and heat stress decrease SOD (Figure 8A) and CAT activity (Figure 8B). Interestingly, cells pretreated with AuNPs show remarkable differences in the level of SOD and CAT activity as compared to cells exposed to cold or heat stress, which seems to be a response to increased intracellular production of O_2_ during heating, because it does not occur in the absence of dioxygen [56]. Thus, it appears that heating increases O_2_ and H_2_O_2_, thereby producing auto-oxidation within *E. coli*, possibly by disruption of the electron transport assemblies of the plasma membrane, and that increased O_2_ production elicits increased biosynthesis of MnSOD [56]. Conversely, Benov and Fridovich showed that, when a mid-log phase culture of the *sodA sodB* strain was incubated at 43 °C, there was a moderate increase in Cu and ZnSOD over 3 h, followed by a steep decline [57]. At 48 °C, a loss was observed due to the thermolabile properties of Cu and ZnSOD. Our results are consistent with the findings of Benov and Fridovich [57].

The freezing process has been shown to generate free radicals in bacterial cells; however, the result depends on the level of available oxygen. The response of aerobically grown *E. coli* cells to cold stress induced by the rapid lowering of growth temperature from 37 to 4 °C was found to be basically the same as the oxidative stress response [54]. The decreased SOD and CAT activity in response to cold and heat stress were interpreted as oxidative, and both cold and heat stresses were attributable to a rise in the intracellular level of superoxide anions [54]. Furthermore, our results are in line with previous findings reporting that cold-shocked cells of *V. cholera* exhibited significantly lower levels of SOD activity than unstressed cells [58]. The data from our study and previous studies demonstrate that the differential responses of SOD and CAT activity are due to the temperature-shifting effect and that these enzymes are regulated by temperature, thus making *E. coli* more susceptible to oxygen radicals. Taken together, AuNPs may act as antioxidative agents that protect *E. coli* from both cold and heat-induced oxidative stress.

## 3. Materials and Methods

### 3.1. Bacteria and Chemicals

The *E. coli* strain used was DH5α from our lab stock. DTNB [5,50-dithio-bis-(-2-nitrobenzoic acid)], and glutathione (GSH) were purchased from Sigma-Aldrich (St. Louis, MO, USA). Luria-Bertani (LB) agar was purchased from USB Corporation (Santa Clara, CA, USA). The HAuCl_4_, *in vitro* toxicology assay kit was purchased from Sigma-Aldrich. BacTiter-Glo™ Microbial Cell Viability Assay Reagent was purchased from Promega (Madison, WI, USA). All other chemicals were purchased from Sigma-Aldrich unless otherwise stated.

### 3.2. Media and Bacterial Growth Analysis

Luria-Bertani (LB) media were prepared and used as previously described [59]. *Bacillus clausii* and *E. coli* cultures were first grown aerobically at 37 °C in LB medium. The cells were harvested by centrifugation, washed twice with phosphate-buffered saline (pH 7.3), and re-suspended in an appropriate fresh medium, such as LB, to the desired initial optical density (absorbance). Inoculated cultures were grown in a shaker (120 rpm) in Erlenmeyer flasks (medium volume/flask volume of 1/10) at 37 °C until they reached the stationary phase. Growth was monitored spectrophotometrically by periodically measuring the absorbance at 600 nm. The bacteria were routinely maintained on LB agar slants or plates at 37 °C and preserved in glycerol stock solutions at −70 °C. Unless otherwise stated, three independent trials were performed for all experiments.

### 3.3. Extracellular Synthesis of AuNPs

Extracellular synthesis of AuNPs was according to Murugan *et al.* [32]. *B. clausii* was inoculated into flasks containing sterile LB broth, and the flasks were incubated at 37 °C for 24 h at 120 rpm. After the incubation period, the culture was centrifuged at 10,000 rpm and the supernatant used for the synthesis of AuNPs nanoparticles. The synthesis was carried out by incubating 1 mM HAuCl_4_ solution with bacterial culture supernatant for 2 h at 40 °C. The extracellular synthesis of AuNPs was monitored by visual inspection of the test tubes for a change in the color of the culture medium from a clear, light yellow to reddish purple, and the synthesis of AuNPs was confirmed by the presence of the AuNPs peak in the UV-vis spectrum.

### 3.4. Characterization of AuNPs

Characterization of synthesized AuNPs particles was carried out according to the methods described previously. The AuNPs were primarily characterized by UV-visible spectroscopy. UV-vis spectra were obtained using an OPTIZEN POP spectrophotometer (Mecasys Co., Seoul, Korea) at Konkuk University, South Korea. The particle size of the dispersions was measured by a Zetasizer Nano ZS90 (Malvern Instruments, Ltd., Malvern, UK). X-ray diffraction (XRD) analyses were carried out on an X-ray diffractometer (Bruker D8 DISCOVER; Bruker AXS, Madison, MA, USA). The high-resolution XRD patterns were measured at 3 kW with a Cu target, using a scintillation counter (λ = 1.5406 Å) at 40 kV and 40 mA, and were recorded in the range of 2θ = 5°–80°. Further characterization of changes in the surface and surface composition was performed by Fourier transform infrared (FTIR) spectroscopy (PerkinElmer Spectroscopy GX, PerkinElmer Inc., Waltham, MA, USA). Transmission electron microscopy (TEM) using a JEM-1200EX microscope (JEOL Ltd, Tokyo, Japan) was performed to determine the size and morphology of the AuNPs. TEM images of AuNPs were obtained at an accelerating voltage of 300 kV. The presence of Au metals in the sample was analyzed by energy-dispersive X-ray analysis (EDX).

### 3.5. Heat and Cold Stress Treatments

For the cold stress treatments, cultures of *E. coli* (OD600 nm = 0.5) were transferred from 37 °C to 4 °C and then incubated for 2 h. For heat stress treatments, cultures of *E. coli* (OD600 nm = 0.5) were transferred from 37 °C to a 45 °C water bath, and then incubated for 2 h. Cells not exposed to cold or heat stress served as controls. The cells grown at 37 °C served as another control. For AuNPs treatments, cells were pre-treated with AuNPs (1 μg/mL) for 1 h and then exposed to the appropriate treatment temperature (37 °C, 4 °C, or 45 °C). The temperature was maintained for 2 h. Subsequent recovery involved return to 37 °C after cold and heat stress. After cold or heat treatment, the cells were removed, the medium was changed, and the cells were allowed to recover from heat stress for 6 or 12 h. Bacterial cell growth was monitored by both CFU and OD_600_ measurement. Growth under cold (4 °C) and heat stress conditions (45 °C) was also examined using the same method. To compare growth under stress with growth under non-stress conditions, the strains were grown at 37 °C in the presence and absence of AuNPs; such conditions are referred to as control conditions throughout this paper. To determine the effect of AuNPs on viability and other biochemical assays, the strains were also grown in the absence of AuNPs at 4 °C and 45 °C without prior pretreatment. All experiments were repeated at least three times.

### 3.6. Bacterial Cell Lysate Preparation

To prepare bacterial cell lysates, cells were grown and centrifuged at 4 °C for 10 min at 5000 rpm. The pellet was then washed with PBS and re-suspended in bacterial lysis buffer, followed by the addition of lysozyme and incubation at 4 °C for 4 h before probe sonication for 5 min. Cell debris was removed by centrifugation at 10,000 rpm, and the supernatant was collected and used for enzyme assay [60].

### 3.7. Effect of AuNPs on E. coli

Assessment of AuNPs on microbial toxicity was performed as described previously [61]. To examine the effect of AuNPs on the growth of *E. coli*, overnight cultures were centrifuged at 6000 rpm for 5 min, washed with 1x PBS, and the pellet re-suspended in saline buffer. Finally, the OD600 of the sample was adjusted to 0.1. The cells (10^6^) were exposed to different concentrations of AuNPs in 96-well round-bottom plates, in triplicate. Bacteria were harvested at the indicated times or dose responses, and the number of CFUs was determined. Medium and medium with AuNPs served as controls. All samples were plated in triplicate, and values of three independent experiments were averaged.

### 3.8. Measurement of Reactive Oxygen Species (ROS)

ROS generation was measured according to the method described earlier, using 2′,7′-dichlorofluorescein diacetate (DCFDA) [43]. Bacterial cells (10^6^ CFU/mL) were treated at the required temperature for 2 h, with AuNPs and without AuNPs. After incubation, the cells were centrifuged at 4 °C for 30 min at 300 × *g*, and each supernatant was treated with 100 μM DCFDA for 1 h. The ROS formed in the sample were detected at 485/20 nm of fluorescence excitation wavelength and 528/20 nm of emission wavelength using a fluorescence multi-detection reader (Bio Tek, Winooski, VT, USA).

### 3.9. Measurement of MDA

The concentration of MDA generated in the culture media from the cells incubated at the required temperature was determined using thiobarbituric acid-reactive substances assay as described previously, with suitable modifications [62]. Briefly, an aliquot of 1 mL of culture media was collected from the treated cells and 10% SDS was added and swirled vigorously. Next, 2 mL of freshly prepared thiobarbituric acid (TBA) was added to the above mix and incubated at 95 °C for 60 min. The reaction was then cooled to room temperature and centrifuged at 5000 rpm for 10 min, and the OD of the supernatant was measured at 530 nm.

### 3.10. Measurement of LDH

For LDH assay, bacterial cells (10^6^ CFU/mL) were treated at the required temperature for 2 h, with and without AuNPs. After incubation, the cells were centrifuged at 4 °C for 30 min at 300× *g*, and then each supernatant was discarded. The pellet was washed twice and treated with LDH reaction solution in a microplate [43]. The plate was incubated with gentle shaking on an orbital shaker for 30 min at room temperature. After incubation, the O.D. of the plate was determined at 490 nm.

### 3.11. Measurement of ATP Levels

Measurement of ATP levels in the bacterial culture supernatant was according to Mempin *et al.* [49] and the manufacturer’s instructions for BacTiter-Glo™ Microbial Cell Viability Assay Reagent (Promega). This is a luciferase-based assay, and the ATP level is determined by measuring luminescence levels and comparing to an ATP standard curve. Briefly, 100 µL of culture supernatant from the control or cells exposed to stress was mixed with an equal volume of BacTiter-Glo™ Microbial Cell Viability Assay Reagent in a 96-well opaque plate and incubated at room temperature for 5 min. After incubation, the luminescence was read in a SpectraMax M2 plate reader (Molecular Devices, Sunnyvale, CA, USA).

### 3.12. Assay for the Leakage of Proteins and Sugars

Protein and sugar leakage in bacterial cells was determined as described previously [63]. Bacterial cells (10^6^ CFU/mL) were treated at the required temperature for 2 h, with and without AuNPs. Each culture was incubated in a shaking incubator at 37 °C for 4 h. Culture samples (1 mL from each culture) were centrifuged at 4 °C for 30 min at 10,000 rpm, and the supernatant was frozen at −20 °C before being used to estimate the protein and sugar levels.

### 3.13. Estimation of GSH Level

To enzymatically determine GSH levels, cells were incubated at the required temperature with or without AuNPs for 2 h. Cells were pelleted by 5-min centrifugation at 10,000 rpm, washed with PBS, and lysed. The lysate was prepared as described above. The amount of GSH was measured enzymatically in the clear supernatant, based on the reduction of 5,5′-dithiobis-(2-nitrobenzoic acid) by the GSH reductase system, as described previously [64]. All samples were prepared in triplicate. DTNB (Ellman’s reagent, 5,50-dithio-bis-(2-nitrobenzoic acid), Sigma-Aldrich) was added to the mixtures to yield a yellow product. The absorbance at 412 nm was measured using a spectrophotometer.

### 3.14. Determination of GST Total Activity

GST activity was followed as described previously [55], with suitable modifications. GST was assayed spectrophotometrically at 37 °C in a mixture containing 900 mL 100 mM potassium phosphate buffer (pH 6.5), 25 mL 40 mM 1-chloro-2,4-dinitrobenzene (CDNB), 50 mL 1 mM GSH, and 25 μL enzyme extract. The reaction mixture was followed by monitoring the increase in absorbance at 340 nm over 5 min. GST activity was expressed as μmol/min/mg protein.

### 3.15. Determination of Superoxide Dismutase and Catalase Activity

Catalase activity was measured using a protocol described previously [65] with suitable modifications. In a typical reaction, a mixture of 500 μL 0.1 M phosphate buffer (pH 7.5) and 500 μL freshly prepared 0.9% (volume percent) H_2_O_2_ solution was prepared. Bacterial cell lysate (100 µL) was added to the mixture and incubated for 3 min. The reaction was terminated by dropwise addition of 2 N H_2_SO_4_. The unreacted H_2_O_2_ was titrated with 0.1 N KMnO_4_. Boiled bacterial cell lysate with or without AuNPs was used as a blank. Enzyme activity was calculated as described previously. SOD activity was measured as described previously [66] using an SOD Assay Kit (Sigma, St. Louis, MO, USA, 19160). Specific enzyme activity was calculated from the enzyme activity and total protein concentration in the bacterial cell lysate, as determined by the Bradford method [60].

## 4. Conclusions

Gold nanoparticles are tremendously useful for diagnostic and therapeutic purposes due to their unique physical, chemical, and biological properties. AuNPs have shown nontoxic and protective effects in several models, including bacteria. However, thus far, no studies have reported the ameliorative effect of AuNPs on oxidative stress induced by cold or heat stress in *E. coli.* Stress includes any parameters that can influence biological systems, and eventually affects viability, growth, metabolism, and reproduction. The stress response of *E. coli* is a complex, vigorous, and adaptable process. Therefore, we first demonstrated the synthesis of AuNPs using a novel bacterium called *Bacillus clausii*. Second, we investigated the antioxidative properties of biologically prepared AuNPs in *E. coli* induced by cold and heat stress. These results of this study indicate that these two different stress responses significantly influence cell viability and metabolic activity as well as the balance of pro- and antioxidant levels at the cellular level. Interestingly, *E. coli* cells pre-treated with AuNPs were able to recover from stress by inducing defense mechanisms, including suppression of ROS and MDA, leakage of proteins and sugars, and increased levels of LDH and ATP. Furthermore, cells pretreated with AuNPs showed increased levels of antioxidants such as GSH, GST, SOD, and CAT. These potential roles of AuNPs in preventing oxidative stress and its adverse effects as induced by cold and heat stress conditions could open up a new avenue for preventing disease caused by stress-tolerant bacteria. In fact, the activation of stress responses is an important factor for antimicrobial resistance development. An increased understanding of the mechanisms and regulation of the stress adaptation of *E. coli* by AuNPs will provide information for pathogenic control, improve the effectiveness of design of novel control methods, and increase the microbial safety of foods. It is a valuable model for therapeutic targets and methods of targeting them using nontoxic, biocompatible, antioxidative agents.
